# A New Strategy to Find Targets for Anticancer Therapy: Chemokine CXCL14/BRAK Is a Multifunctional Tumor Suppressor for Head and Neck Squamous Cell Carcinoma

**DOI:** 10.5402/2012/797619

**Published:** 2012-11-14

**Authors:** Ryu-Ichiro Hata

**Affiliations:** Oral Health Science Research Center, Kanagawa Dental College, 82 Inaoka-cho, Yokosuka 238-8580, Japan

## Abstract

In order to find a suppressor(s) of tumor progression *in vivo* for head and neck squamous cell carcinoma (HNSCC), we searched for molecules downregulated in HNSCC cells when the cells were treated with epidermal growth factor (EGF), whose receptor is frequently overactivated in HNSCC. The expression of BRAK, which is also known as CXC chemokine ligand 14 (CXCL14), was downregulated significantly by the treatment of HNSCC cells with EGF as observed by cDNA microarray analysis followed by reverse-transcriptase polymerase chain reaction analysis and western blotting. The EGF effect on the expression of CXCL14/BRAK was attenuated by the copresence of inhibitors of the EGF receptor, MEK, and ERK. The rate of tumor formation *in vivo* of BRAK-expressing vector-transfected tumor cells in athymic nude mice or SCID mice was significantly lower than that of mock vector-transfected ones. In addition tumors formed *in vivo* by the BRAK-expressing cells were significantly smaller than those of the mock-transfected ones. These results indicate that CXCL14/BRAK is a chemokine having suppressive activity toward tumor progression of HNSCC *in vivo*. Our approach will be useful to find new target molecules to suppress progression of tumors of various origins in addition to HNSCC.

## 1. Introduction

Head and neck cancer is the sixth most common cancer worldwide. The most common type of head and neck cancer is squamous cell carcinoma (HNSCC); disappointingly, despite advances in surgical and other treatments that enhance quality of life and palliative value, survival rates are not improving for this cancer. HNSCC is a collective term for cancers at several sites (for example, the oral cavity, pharynx, and larynx) that have different etiologies and prognoses, even though they share some risk factors, including tobacco smoking and alcohol consumption and by infection with high-risk types of human papilloma virus [7, 8].

Tumors develop in multiple steps [9–11], and tumor progression is dependent on the balance of the expression between tumor progression-promoting and -suppressing genes being in favor of the former at each step [12, 13]. In order to prevent tumor progression, many investigators have searched for molecules that are overexpressed during tumor progression as target molecules for therapeutic drugs and have tried to prevent tumor progression by inhibiting these tumor-promoting molecules. However, drugs for many of the target molecules were not successful for clinical applications owing to the serious side effects of these drugs, which is not surprising because these target molecules are also important for normal development and maintenance of tissues and for homeostasis of human body [14, 15]. On the other hand, activation of presumptive tumor suppressor(s) or inhibition of its downregulation may be much more promising for the prevention of tumor progression without significant side effects, because these molecules are supposedly present abundantly in normal tissues. In the course of our study to find an endogenous tumor suppressor(s) for HNSCC, we searched for molecules downregulated in HNSCC cells, when the cells were treated with epidermal growth factor (EGF), whose receptor is frequently overactivated in HNSCC and found significant downregulation of certain genes. Here we describe our data to find *in vivo *tumor suppressor for HNSCC and applications for suppression of growth of tumors from various origins. 

## 2. The BRAK Box Is Opening 

### 2.1. Chemokine CXCL14/BRAK Is an Intercellular Tumor Suppressor for HNSCC

**Figure 1:**
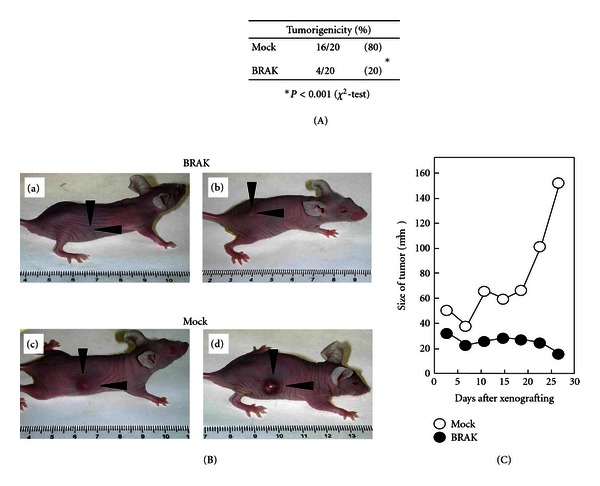
CXCL14/BRAK expression in HNSCC cells suppresses growth of tumor cell xenografts in athymic nude mice. (A) BRAK-expressing tumor cells or mock vector-transfected ones were injected subcutaneously and tumor sizes were consecutively measured. Tumors were regarded as suppressed, when there were only fat tissues and/or scar tissues observed and no tumor cells were found by histological examination after dissection at 27 days. (B) Photographs of tumor cell xenografts after 27 days. BRAK-expressing cell clones or mock vector-transfected cells (5 × 10^6^) were inoculated subcutaneously into both sides of the back region of 10 female athymic nude mice, representative animals were photographed 27 days after xenografting. (a), (b) BRAK-expressing cells. (c), (d) Mock vector-transfected cells. (C) Mock: open circles, average of 16 tumors from 8 animals. BRAK: closed circles, average of 4 tumors with 4 animals. Two independent experiments showed a quite similar result. Difference between mock-transfected and BRAK-expressing cells are shown. Significant difference in the sizes of tumors was observed (Cited from [1]).

**Figure 2:**
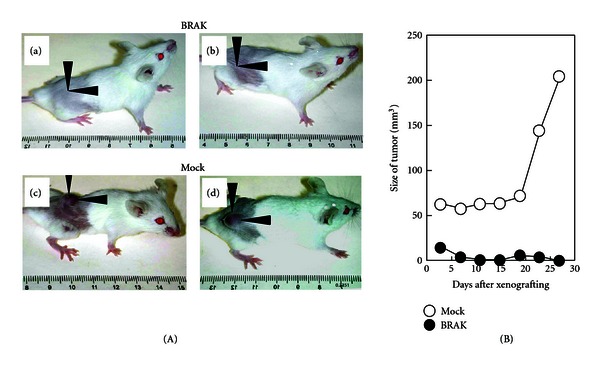
CXCL14/BRAK expression in oral carcinoma cells (HSC-3) completely suppresses growth of tumor cell xenografts in SCID mice. (A) Photographs of tumor cell xenografts after 27 days. BRAK-expressing cell clones or mock vector-transfected cells (5 × 10^6^) were inoculated subcutaneously into both sides of the back region of 10 female SCID mice, representative animals were photographed 27 days after xenografting. (a), (b) BRAK-expressing cells. (c), (d) Mock vector-transfected cells. (B) Effect of BRAK expression on HSC-3 tumor xenografts in SCID mice. Pooled clones of BRAK-expressing (BRAK) and mock vector-transfected (Mock) cells (5 × 10^6^/site) were inoculated subcutaneously into both sides of the back region of 10 female SCID mice. Mock: open circles, average of 20 tumors in 10 animals. BRAK: closed circles, average of 20 tumors in 10 animals. A significant difference in the size of tumors was observed at all points measured. *P* < 0.001 (Student's *t*-test). (Cited from [2]).

**Figure 3:**
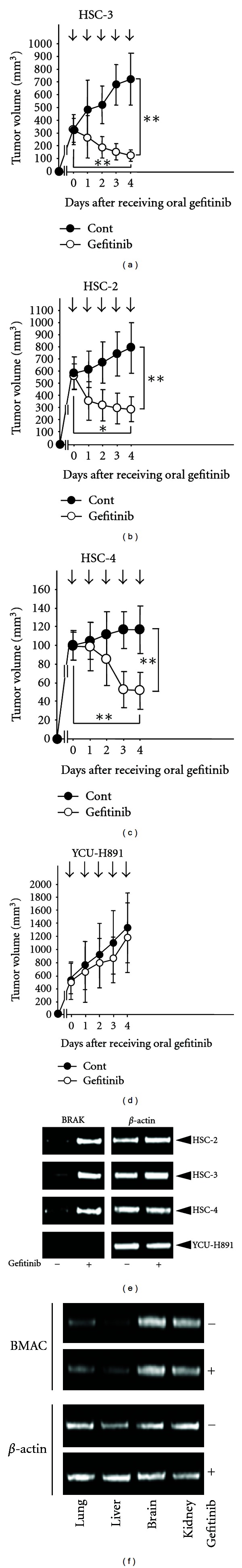
Effects of oral gefitinib on the tumor volume and BRAK or BMAC mRNA level. HNSCC cells (1 × 10^7^) of lines HSC-2 (a), HSC-3 (b), HSC-4 (c), and YCU-H891 (d) were subcutaneously injected separately into both flanks of 10 female athymic nude mice and allowed to form a tumor burden for 10 days. The mice were daily-administered oral gefitinib (50 mg/kg/day). Tumor volume was measured daily. In some mice treated with gefitinib for 4 days, tumor tissues or host organs were taken to extract total RNA. Human CXCL14/BRAK or mouse BMAC mRNA expressions in transplants (e) and host organs (f) were determined by RT-PCR. PCR products were visualized by ethidium bromide staining after electrophoresis in 1.5% agarose gel. Arrows indicate time-point of administration of gefitinib. **P < 0.001 (Student's *t*-test); values are expressed as the mean ± SD (*n* = 10) (Cited from [3]).

Chemokines are a group of small proteins with molecular masses in the range of 8 to 12 kDa, and they are mostly basic and structurally related molecules that are reported to regulate cell trafficking of various types of leukocytes through interaction with a subset of seven-transmembrane, G protein-coupled receptors [16–19]. Chemokine domains are defined by the presence of four conserved cysteine residues linked by two disulfide bonds. Two major subfamilies, CXC and CC chemokines, are distinguished according to the position of the first two cysteine residues, which are separated by one amino acid (CXC subfamily, Table 1) or are adjacent to each other (CC subfamily) [19, 20]. BRAK/CXCL14 (breast- and kidney-expressed chemokine/CXC chemokine ligand 14) is a non-ELR (Glu-Leu-Arg) CXC chemokine and is expressed ubiquitously and constitutively in epithelia throughout the body [21] and several physiological functions of it have been proposed, such as recruitment and maturation of monocyte-derived macrophage and renewal of Langerhans cells in the skin [22, 23]. Promotion of trafficking of natural killer cells to the sites of inflammation [24] and macrophage infiltration into white adipose tissue in obese mice fed a high-fat diet [25], as well as inhibition of angiogenesis [26], were also reported as functions of this chemokine. We found that expression of BRAK/CXCL14 is downregulated significantly by the treatment of HNSCC cells with EGF as observed by cDNA microarray analysis (Table 2) [1] followed by reverse-transcriptase polymerase chain reaction analysis. In order to investigate whether CXCL14/BRAK has a tumor-suppressing effect *in vivo*, we prepared CXCL14/BRAK-expression vector-transfected and mock vector-transfected tongue tumor cells. The rate of *in vivo* tumor formation by BRAK-expressing vector-transfected tumour cells in athymic nude mice (Figure 1) or SCID (Figure 2) mice was significantly lower than that of mock vector-transfected ones; and, in addition, the tumors formed *in vivo* by these BRAK-expressing cells are significantly smaller than those of the mock vector-transfected ones [1, 2]. Interestingly, the oral administration of gefitinib, an inhibitor of EGF receptor, significantly (*P* < 0.001) reduces tumor growth of xenografts of three HNSCC cell lines (HSC-2, HSC-3, and HSC-4) in female athymic nude mice accompanied by an increase in CXCL14/BRAK expression specifically in the tumor tissue (Figures 3(a), 3(b), 3(c), and 3(e)). This tumor-suppressing effect of the drug is not observed in the case of CXCL14/BRAK nonexpressing YCU-H891 cells (Figures 3(d), and 3(e)). Furthermore, the introduction of a CXCL14/BRAK short hairpin shRNA reduces both the expression levels of CXCL14/BRAK in HSC-3 cells and the antitumor efficacy of gefitinib *in vivo* [3]. These results indicate that CXCL14/BRAK is a chemokine having suppressive activity toward tumor progression of HNSCC *in vivo*. The data are also well coincided with lower expression levels of CXCL14/BRAK in HNSCC tissues than in adjacent normal tissue [26].

### 2.2. CXCL14/BRAK Expression in HNSCC Cells Reduces Both the Rate of Settlement and Proliferation of the Cells *In Vivo* after Settlement of the Cells

**Figure 4:**
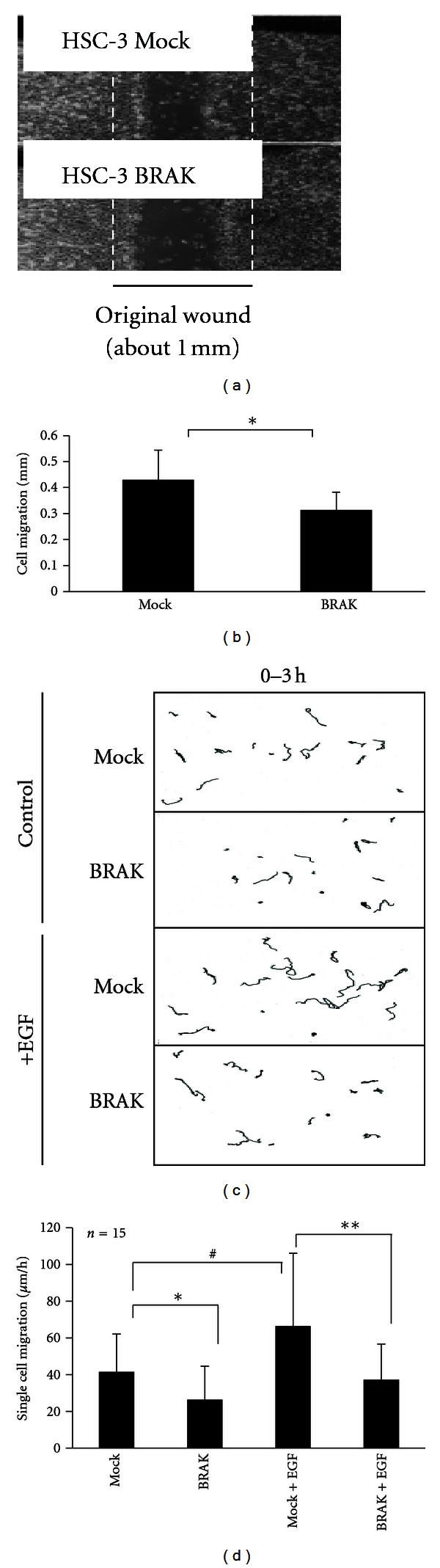
CXCL14/BRAK expression in carcinoma cells reduces cell motility. (a) Wound-healing (scratch) assay. Confluent HSC-3 Mock or HSC-3 BRAK cells grown in 6-well plates with DMEM containing 10% FBS were scratched with the 200 *μ*L tip to make an approximate 1 mm wide wound; and then the wells were washed with fresh medium to remove cell debris, and the plates were further incubated for 6 h. Cell images of 3 identical fields at 0 h and 6 h for each sample were obtained by phase-contrast microscopy, and the photos at 6 h are shown. White dashed lines indicate original width of the wound. (b) The cell migration was determined in triplicate by use of ImageJ software, and the average of the migration distance in each sample was calculated. Statistical significance was analyzed by Student's *t*-test calculated from the total data obtained from 3 independent experiments. (c) Cell nuclear trajectory line for 3 h in the absence (control) or in the presence of 100 pg/mL of recombinant EGF is shown. (d) The migration rates were determined by ImageJ software. *P < 0.05, **P < 0.02. (Cited from [4]).

The forced expression of CXCL14/BRAK in tongue carcinoma cells decreases the rate of tumor formation and size of tumor xenografts in athymic nude mice [1] and SCID mice [2] as described above. In these experiments on cloned cells with upregulated CXCL14/BRAK protein expression, the growth of these cells under culture conditions is the same as those of control mock vector-transfected cells. However, the migration rate of the CXCL14/BRAK-expressing cells *in vitro* is significantly slower than that of the mock-vector transfected cells (Figure 4); and the attachment of the cells to collagen is much faster than the control cells [4]. 

Recent progress in cancer research has shown that cancerous tissues *in vivo* are derived from colonies of cancer stem cells [27–29]. These data have raised 3 possibilities regarding the apparent slower growth rate of xenografted CXCL14/BRAK-expressing tumor cells. The first is that the ratio of stem cell-like cells among the CXCL14/BRAK-expressing cells is smaller, and thus a smaller number of carcinoma cells settle in the tissues of the host mice. The second possibility is that the growth rate of CXCL14/BRAK-expressing cells *in vivo* is slower than that of mock-vector transfected cells. The third one is both the rate of settlement and proliferation of the cells *in vivo* after settlement of the cells is reduced.

**Figure 5:**
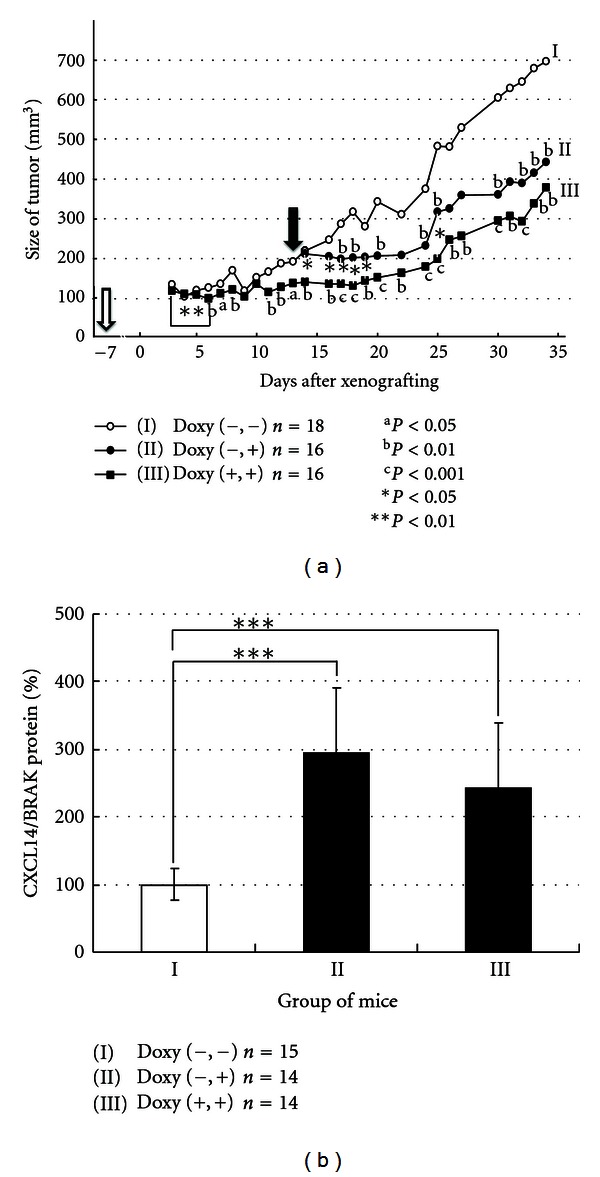
Expression of CXCL14/BRAK protein in HNSCC cells suppresses growth of the cells *in vivo*. (a) Tumor size of xenografted Tet-on BRAK HSC-2 cells. The mice were subcutaneously injected with 10^7^ Tet-on BRAK HSC-2 cells, and the animals were provided 5% sucrose-containing water (Group I). Other mice were given sucrose solution containing 2 mg/mL doxycycline starting at 13 days (black arrow) after implantation of the tumor cells (Group II). A third group of mice were implanted with Tet-on BRAK HSC-2 cells that had been precultured in the presence of 0.1 *μ*g/mL of doxycycline for 7 days (Group III). These mice were provided doxycycline-supplemented water 7 days before (white arrow) implantation of the tumor cells. ^a^ *P* < 0.05; ^b^ *P* < 0.01; ^c^ *P* < 0.001 between group I and II or III. *P < 0.05 between Group II and III and **P < 0.01 between day 3 and day 6 of Group III. (b) Expression levels of CXCL14/BRAK proteins in transplanted tumor cells *in vivo* 35 days after transplantation, as determined by western blotting. ***P < 0.001, as indicated by the bracket. (Cited from [5]).

In order to clarify whether the expression of CXCL14/BRAK affects the settlement of carcinoma cells in host tissues *in vivo* or proliferation of the colonized carcinoma cells or both, we prepared oral floor carcinoma-derived HSC-2 cells in which CXCL14/BRAK expression was induced upon doxycycline treatment. Then 30 nude mice were separated into three groups composed of 10 mice per group: Group I, the control, in which the engineered cells were directly xenografted onto the back of the mice; Group II, the cells were xenografted and then the mice were treated with doxycycline; and Group III, the cells were pretreated with doxycycline during culture, and the host mice were also treated with the drug before and after xenografting. The effects of CXCL14/BRAK expression were examined by measuring the tumor size. The order of the size of tumor xenografts was I > II > III, even though the growth rate of the engineered cells is the same whether or not the cells were cultured in the presence of the drug [5]. In addition, the size of tumors is significantly downregulated after xenografting the doxycycline-pretreated cells in Group III. These data indicate that CXCL14/BRAK expression in oral floor carcinoma cells reduces both the rate of settlement and the proliferation of the cells *in vivo* after settlement of the cells (Figure 5) [5].

### 2.3. Paracrine and Endocrine Function of Chemokine CXCL14/BRAK

Our data indicate that expression of CXCL14/BRAK in tumor cells suppresses tumor growth *in vivo* by acting in an autocrine or paracrine fashion [1, 2]. 

On the other hand heightened CXCL14/BRAK expression has been reported to occur in adenocarcinomas such as prostate [30] and breast [31, 32] cancers and in pancreatic cancer cells [33]. These data suggest that effects of CXCL14/BRAK on development and progression of cancer may be quite different between HNSCC and adenocarcinoma. In prostate cancer, CXCL14/BRAK mRNA is significantly upregulated in localized prostate cancer and its level positively correlates with the Gleason score [30]. However, interesting enough, overexpression of normal CXCL14/BRAK in prostate cancer cells by introducing mouse or human CXCL14/BRAK expression vectors retards tumor growth *in vivo* compared with the growth of control vector-transfected tumor cells [30]. These data suggest the possibility that expression of nonfunctional CXCL14/BRAK molecules might be associated with stimulation of tumor growth in several adenocarcinoma cells, whereas normal functioning CXCL14/BRAK molecules might suppress tumor growth *in vivo* in adenocarcinoma as well as in HNSCC. 

In order to investigate whether CXCL14/BRAK suppresses the growth of tumor cells of other tissue origins in a paracrine or endocrine fashion, we produced CXCL14/BRAK transgenic (Tg) mice and examined the growth of tumor cell transplants in them. Beta-actin promoter-regulated CXCL14/BRAK cDNA was introduced into male C57BL/6J pronuclei, and three lines of Tg mice, which produced an approximately 10 times higher than normal amount of CXCL14/BRAK protein in their blood, were obtained. These Tg mice show suppressed growth of Lewis lung carcinoma (LLC) cell and B16 melanoma cell-transplants, indicating CXCL14/BRAK, first found as a tumor progression suppressor for HNSCC, also suppresses the progression of tumors of other tissue origins by a paracrine or endocrine mechanism [6]. 

**Figure 6:**
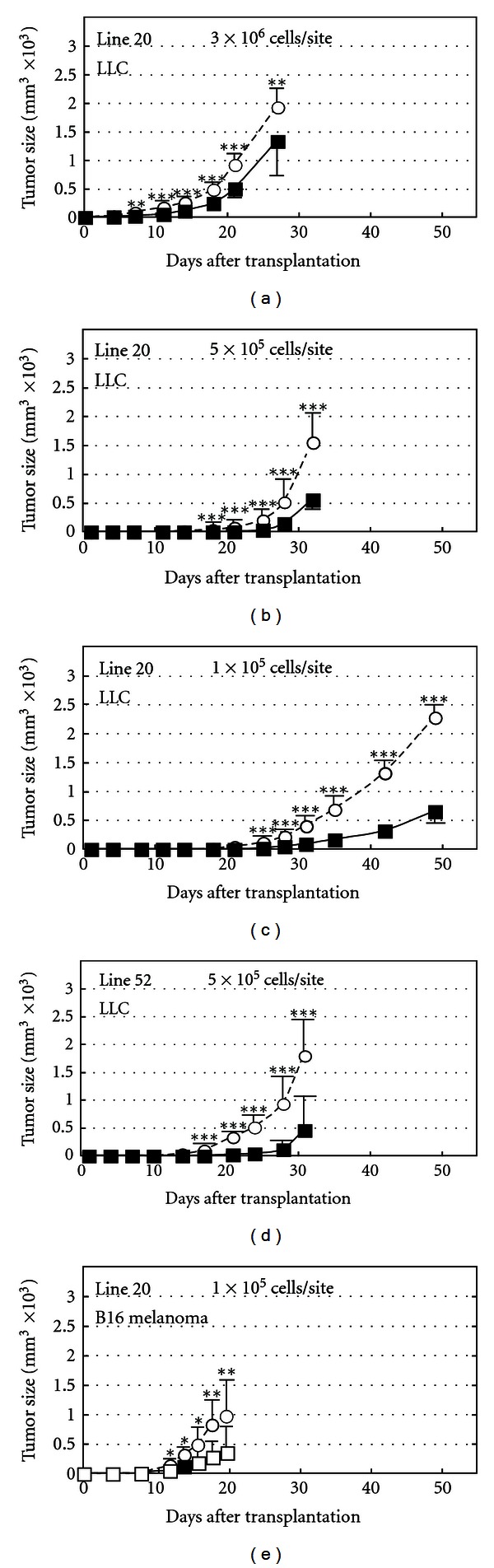
Growth suppression of transplants of Lewis lung carcinoma (LLC) cells and melanoma cells in CXCL14/BRAK transgenic mice. Various concentrations of tumor cells per 200 micro liter of phosphate buffered saline were injected both sides of each of 10 transgenic (Tg) or wild type (Wt) mice, and tumor size was measured. (a), (b), (c) LLC cells in Line 20 mice, (d) LLC cells in Line 52 mice. (e) melanoma cells in Line 20 mice. **P < 0.01, ***P < 0.001. (Cited from [6]).

Significant growth suppression of LLC tumor cell transplants was observed in 3 independent lines of CXCL14/BRAK Tg mice (Figure 6 and unpublished data), indicating that tumor suppression was due to the high expression of CXCL14/BRAK in the Tg mice and not to the destruction of putative tumor progression stimulator, which might be present in wild-type mice. We could not detect expression of mouse CXCL14/BRAK gene in either LLC or melanoma cells by RT-PCR, indicating that CXCL14/BRAK produced by the Tg mice functions in a paracrine and/or endocrine fashion [6]. 

**Figure 7:**
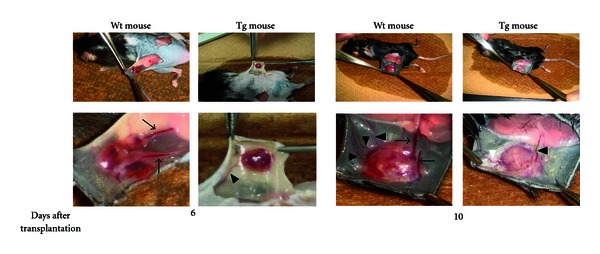
Difference in vascular structure of tumor transplants between Wt and Tg mice. Mice were dissected under ether anesthesia and tumor vasculatures were photographed. Arrows indicate blood vessels larger than 400 *μ*m in diameter and arrowheads those smaller than 100 *μ*m in diameter. (Cited from [6]).

We observed suppression of blood vessel penetration into tumors of Tg mice (Figure 7), suggesting CXCL14/BRAK suppresses angiogenesis, as would be anticipated by the absence of ELR motif in the N-terminal part of the molecule. It is reported that recombinant CXCL14/BRAK inhibits *in vivo *angiogenesis induced by IL-8 (CXCL8), basic FGF or VEGF but that binding of CXCL14/BRAK to human umbilical vein endothelial cells and human dermal microvascular endothelial cells could not be detected [26]. Our data indicate inhibition of penetration of smooth muscle cell actin-positive cell into tumors of Tg mice in addition to inhibition of vascular endothelial cells into tumors [6]. Thus, CXCL14/BRAK might inhibit penetration of vasculature into tumors by inhibiting chemotaxis of perivascular smooth muscle cells and formation of a mature functional microvasculature. 

 The suppression of tumor growth is attenuated by the injection of anti-NK cell antibodies indicating that NK cell activity is also essential for the suppression of tumor cell growth in these Tg mice (Hata et al., unpublished data).

**Figure 8:**
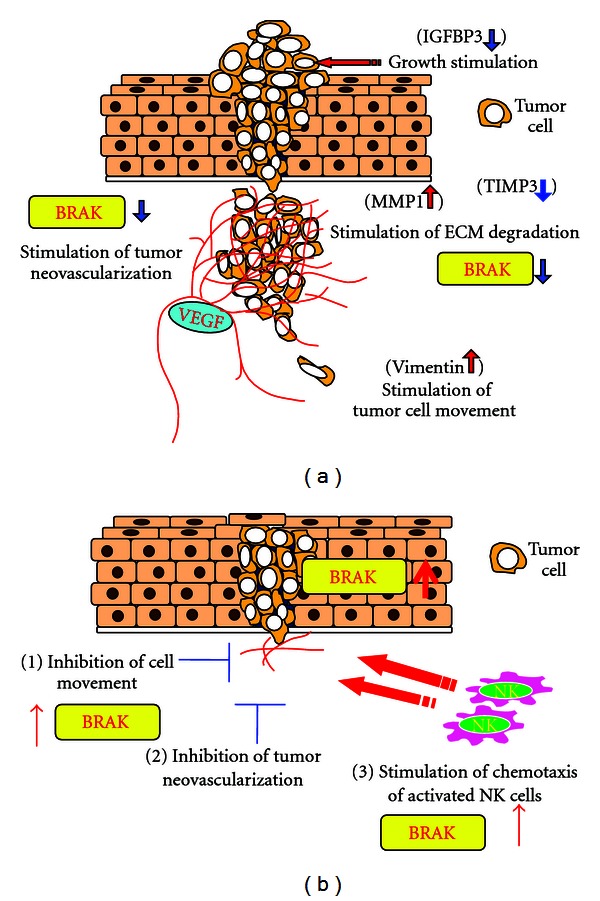
EGF downregulates expression of CXCL14/BRAK, a multifunctional tumor suppressor. (a) EGF stimulates tumor progression by regulating expression of tumor-progression factors in addition to CXCL14/BRAK. Upward red arrows indicate upregulation of the gene expression and downward blue arrows indicate downregulation of the gene expression. (b) CXCL14/BRAK is a multifunctional tumor suppressor.

These data also indicate that BRAK/CXCL14 is a multifunctional tumor suppressor (Figure 8).

### 2.4. Regulation of Expression of Chemokine CXCL14/BRAK

**Figure 9:**
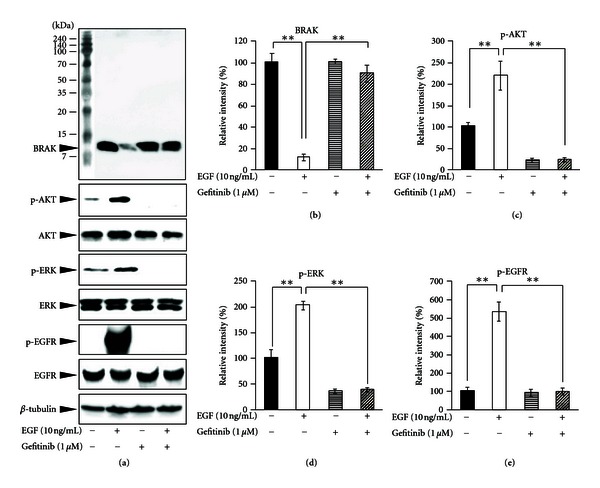
Gefitinib restores epidermal-growth-factor (EGF-)induced CXCL14/BRAK repression with a concomitant decrease in the level of phosphorylated EGF receptor (EGFR) and extracellular signal-regulated kinase (ERK). Nearly confluent HSC-3 cells were incubated with or without gefitinib (1 *μ*M) and/or EGF (10 ng/mL). (a) Cells were incubated with EGF for 15 min for detection of EGF receptor (EGFR), phosphorylated EGFR (pEGFR), ERK, phosphorylated ERK (pERK), Akt and phosphorylated Akt (pAKT) levels and for 24 h to detect the BRAK protein level. Their protein levels were determined by western blotting after treatment with respective antibodies. Relative intensities for BRAK (b) and phosphorylated levels of Akt (pAKT, (c)), ERK (pERK, (d)), and EGFR (pEGFR, (e)) were normalized by *β*-tubulin and their total proteins, respectively. **P < 0.001 (Student's *t*-test); values are presented as mean ± SD (*n* = 3 in all panels). (Cited from [3]).

We have shown that EGF downregulates CXCL14/BRAK mRNA expression [1]. Moreover, this downregulation was blocked by the copresence of PD98059, a specific inhibitor for MEK, suggesting that the EGFR-MEK-ERK signaling pathway is involved in EGF-induced BRAK downregulation [3]. In order to examine this possibility, we investigated dose-dependent effects of several enzyme inhibitors on CXCL14/BRAK mRNA expression. In the presence of 10 ng of EGF, the EGFR inhibitor, gefitinib (Figure 9), MEK inhibitors that act with different mechanisms, PD98059l and U0126, and the ERK inhibitor FR180204 dose-dependently restored expression of CXCL14/BRAK mRNA, thus indicating that the EGFR-MEK-ERK pathway regulates CXCL14/BRAK mRNA expression [3]. Next we examined whether modulation of CXCL14/BRAK mRNA expression by EGF and/or gefitinib is reflected in protein levels of CXCL14/BRAK and whether gefitinib treatment attenuates the EGF effect by elevating the CXCL14/BRAK protein level. In these experiments 1 *μ*M gefitinib was employed, because this concentration is the lowest concentration that induces nearly the maximum effect to induce CXCL14/BRAK. Western blot analysis clearly showed EGF-induced CXCL14/BRAK repression and restoration of this downregulation by gefitinib at the protein level (Figure 9) in concordance with the results obtained by reverse-transcriptase polymerase chain reaction analysis.

It is known that EGFR signaling occurs *via* two major pathways: Ras-Raf-1-MEK-ERK signaling and PI3 kinase-Akt signaling [3]. Our model also showed that EGF treatment stimulates these two major downstream pathways (Figure 9) [3]. Our data showed that the MEK-ERK signaling pathway is involved in EGF-induced CXCL14/BRAK repression. By performing western blot analysis to detect target proteins, we also tested whether the PI3 kinase-Akt signaling pathway participates in the downregulation of CXCL14/BRAK expression and found that the inhibition of PI3 kinase activity by treatment with LY294002 reduces EGF-induced downregulation of phosphorylated Akt (pAKT), but that the LY294002 treatment does not restore EGF suppression of CXCL14/BRAK mRNA expression [3]. These results indicate that MEK-ERK is the major downstream pathway of EGFR in the downregulation of CXCL14/BRAK mRNA expression in HNSCC cells.

**Figure 10:**
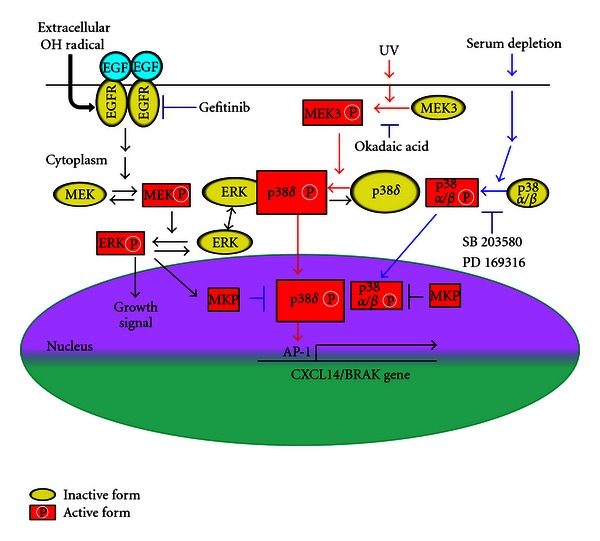
Regulation of CXCL14/BRAK gene expression by the cross talk of MAP kinase subtypes. MKP: MAP kinase phosphatase. Arrows indicate stimulation signals and T-bars indicate inhibitory signals. Red arrows indicate signals stimulated by UV radiation and blue ones indicate those stimulated by serum depletion.

The mitogen-activated protein kinase (MAPK) family comprises ERK, JNK, p38, and ERK5 (big-MAPK, BMK1). UV irradiation of squamous cell carcinoma cells induced upregulation of gene expression of chemokine CXCL14/BRAK, stimulated p38 phosphorylation, and downregulated the phosphorylation of ERK [34]. Human p38 MAPKs exist in 4 isoforms: p38*α*, *β*, *γ*, and *δ*. This UV stimulation of p38 phosphorylation was not inhibited by the presence of SB203580 or PD169316, which are inhibitors of p38*α* and *β* suggesting that p38 phosphorylation is not dependent on these two isoforms and that p38*γ* and/or *δ* is responsible for the phosphorylation. In fact, inhibition of each of these four p38 isoforms by the introduction of short hairpin (sh) RNAs for respective isoforms revealed that only shRNA for p38*δ* attenuates the UV-induced upregulation of BRAK/CXCL14 gene expression. In addition, overexpression of p38 isoforms in cells showed the association of p38*δ* with ERK1 and 2, concomitant with downregulation of ERK phosphorylation. The usage of p38*δ* isoform by the UV-irradiated cells is not merely due to the abundance of this p38 isoform in HSC-3 cells. Because serum deprivation of the cells also induces an increase in CXCL14/BRAK gene expression, and in this case p38*α* and/or *β* isoform is responsible for this upregulation of BRAK/CXCL14 gene expression as observed by the inhibition with SB203580 or PD169316 (Figure 10) [34].

We also observed that oxidative stress induced by H_2_O_2_ or HO^∙^ downregulates gene expression of CXCL14/BRAK via stimulating EGFR/MEK/ERK signaling pathway in human HNSCC cells (Figure 10) [35]. Taken together, the data indicate that the respective stress-dependent action of p38 isoforms is responsible for the upregulation of the gene expression of the chemokine CXCL14/BRAK (Figure 10) [36, 37].

## 3. Conclusion: Chemokine CXCL14/BRAK Is a Hopeful Molecule for Tumor Suppression and Prevention without Side Effects

It was reported that inhibitors of angiogenesis show serious side effects such as stimulation of tumor invasion and metastasis [38, 39]. CXCL14/BRAK expression in tumor cells also suppresses tumor cell mobility [4]; our preliminary data indicate that CXCL14/BRAK Tg mice also suppressed tumor metastasis (Hata et al., unpublished data), thus suggesting that mechanism of inhibition of tumor suppression by CXCL14/BRAK is quite different from that of other reported angiogenesis inhibitors. CXCL14/BRAK-expressing Tg mice show no apparent abnormality when observed up to 2 years of age [6]; and, interestingly, in a normal human population one person was found to express a 10 times higher than normal amount of blood CXCL14/BRAK protein without any apparent abnormalities [40]. These data support the possibility that CXCL14/BRAK expressed at a high level does not have severe side effects.

In conclusion, our data indicate that CXCL14/BRAK Tg has suppressed growth of LLC and B16 melanoma cell transplants. The precise molecular mechanisms of the tumor suppression are not clear at present; our data suggest CXCL14/BRAK is a multifunctional tumor suppressor. The data also indicate that CXCL14/BRAK, which was first found as tumor progression suppressor *in vivo* for HNSCC cells, also suppresses tumors derived from other tissues. Thus CXCL14/BRAK may be a very promising molecular target for tumor suppression without side effects.

## Figures and Tables

**Table 1:** CXC Chemokine ligands and their receptors.

Systematic name	CXC Chemokine		Chemokine receptor	Function
	RowSpanEmpty	Human			RowSpanEmpty	Mouse
ELR + chemokines				
CXCL1	MGSA-*α*/GRO-*α*	GRO/MIP-2	CXCR2 > CXCR1	P
CXCL2	MGSA-*β*/GRO-*β*	GRO/MIP-2	CXCR2	P
CXCL3	MGSA-*γ*/GRO-*γ*	GRO/MIP-2	CXCR2	P
CXCL5	ENA-78	GCP2/LIX?	CXCR2	P
CXCL6	GCP-2	GCP2/LIX?	CXCR1, CXCR2	P
CXCL7	NAP-2	CRA-a,b	CXCR2	P
CXCL8	IL-8	Unknown	CXCR1, CXCR2	P

ELR − chemokines				
CXCL4	PF-4	PF-4	CXCR3A and B	A/S
CXCL9	Mig	Mig	CXCR3	A
CXCL10	IP-10	IP10CRG2	CXCR3	A
CXCL11	I-TAC	I-TAC	CXCR3	A
CXCL12	SDF-1*α*/*β*	SDF1/PBSF	CXCR4, CXCR7	M, P
CXCL13	BCA-1	BLC	CXCR5	?
CXCL14	BRAK	BRAK (BMAC)	?	A/N/S
CXCL16	SR-PSOX	CXCL16	CXCR6	NT/S

A: angiostatic, M: metastatic, N: NK cell regulation, NT: NKT cell regulation, P: tumor progression, S: tumor suppression.

**Table 2:** Regulation of gene expression of tumor-related genes by epidermal growth factor (EGF).

Name	Control	+EGF	Ratio-1	+EGF	Control	Ratio-2	1/Ratio-2
	RowSpanEmpty	Cy3		Cy5	RowSpanEmpty			Cy3	Cy5	RowSpanEmpty
MMP1	2240	10470	4.67	2673	579	0.22	4.61
Vimentin	4596	16877	3.67	5170	1896	0.37	2.73
TIMP3	2755	2113	0.77	631	1404	2.22	0.45
IGFBP3	6081.20	1361	0.22	401	1643	4.09	0.24
BRAK	7174	2089	0.29	654	4637	7.09	0.14

Total RNA was extracted with TRIzol reagent (Invitrogen) from cells treated with EGF for 24 h and untreated control cells and fluorescent cDNAs were prepared separately by a single round of reverse transcription in the presence of fluorescent Cy3-deoxy UTP for EGF-treated cells and Cy5-deoxy UTP for untreated ones. Cy3-deoxy UTP- and Cy5-deoxy UTP-labeled cDNAs were mixed and hybridized with IntelliGene Human Cytokine CHIP Ver. 3.0 (TAKARA). cDNA microarray analysis was performed according to the manufacturer's protocol. The same experiment was repeated by reversing the labels, that is, using Cy5-deoxy UTP for EGF-treated cells and Cy3-deoxy UTP for the untreated ones. The hybridized chips were analyzed by ScanArray 4000 (Perkin-Elmer, Wellesley, MA, USA). MMP1: matrix metalloproteinase-1, TIMP3: tissue inhibitor of matrix metalloproteinase 3, IGFBP3: insulin-like growth factor-binding protein 3.
